# Optimal fracture prediction thresholds for therapy onset, established from FRAX and Garvan algorithms: a longitudinal observation of the population representative female cohort from the RAC-OST-POL Study

**DOI:** 10.1007/s11657-023-01346-3

**Published:** 2023-11-16

**Authors:** W. Pluskiewicz, A. Werner, M. Bach, P. Adamczyk, B. Drozdzowska

**Affiliations:** 1 Department and Clinic of Internal Diseases, Diabetology, and Nephrology, Metabolic Bone Diseases Unit, Faculty of Medical Sciences in Zabrze, Medical University of Silesia in Katowice, 3-Maja 13/15 Street, 41-800 Zabrze, Poland; 2 Department of Applied Informatics, Silesian University of Technology, 44-100, Gliwice, Poland; 3 Department of Pediatrics, Faculty of Medical Sciences in Katowice, Medical University of Silesia in Katowice, Katowice, Poland; 4 Department of Pathomorphology, Faculty of Medical Sciences in Zabrze, Medical University of Silesia in Katowice, Katowice, Poland

**Keywords:** Fracture, Fracture prediction, Osteoporosis, Women

## Abstract

***Summary*:**

The study shows that the use of unified cutoff thresholds to identify high fracture risks by two popular calculators—FRAX and Garvan—leads to a significant discrepancy between the prediction of fractures and their actual prevalence over the period of 10 years. On the basis of the ROC analyses, a proposal of differentiated thresholds is presented. They were established at 6% for FRAX major fracture risk, 1.4% for FRAX hip fracture risk, 14.4% for Garvan any fracture risk, and 8.8% for Garvan hip fracture risk.

**Purpose/introduction:**

The aim of the study was to verify how much were the tools, designed to predict fracture risks, precise vs. the actual fracture incidence values over a prospective observation.

**Methods:**

The study group consisted of a population-based postmenopausal sample from the RAC-OST-POL Study. At baseline, there were 978 subjects at the mean age of 66.4 ± 7.8 years and, after a 10-year follow-up, 640 women remained at the mean age of 75.0 ± 6.95 years. At baseline, the fracture risk was established by the FRAX and Garvan tools.

**Results:**

During the observation period, 190 osteoporotic fractures were identified in 129 subjects. When high-risk fracture cutoff thresholds (of 10% for major/any and 3% for hip fractures) were employed, only 19.59% of major fractures and 50% of hip fractures were identified in the high-risk group. For the Garvan tool, the percentage of correctly predicted fractures for any and hip fractures was 86.05% and 71.43%, respectively. Nevertheless, the fracture prediction by the Garvan tool was associated with the qualification of numerous subjects to the high-risk group, who subsequently did not experience a fracture in the 10-year follow-up period (false-positive prediction). Based on the ROC analyses, new high-risk thresholds were proposed individually for each calculator, improving the sensitivity, specificity, and diagnostic accuracy of these tools. They were established at 6% for FRAX major fracture risk, 1.4% for FRAX hip fracture risk, 14.4% for Garvan any fracture risk, and 8.8% for Garvan hip fracture risk.

**Conclusions:**

The current prospective study enabled to establish new, optimal thresholds for therapy initiation. Such a modified approach may enable a more accurate identification of treatment requiring patients and, in consequence, reduce the number of new fractures.

## Introduction

Fractures are crucial symptoms of osteoporosis, which is usually a process without apparent clinical symptoms, being often called a “silent bone thief.” A typical osteoporotic fracture is a low-trauma fracture after a fall from a standing height. The most common osteoporotic fractures affect the hip, the spine, the forearm, and the arm, and are thus classified as major osteoporotic fractures. Every fracture causes serious health problems for affected patient, including pain, functional impairment, and the necessity of long-term medical care, while hip and spine fractures are especially serious in their effects. Fractures in the two locations may cause permanent functional deterioration and also terminate patient’s life; a considerable number of subjects die during the first months after hip/spine fracture [1]. One should also remember that a prior fracture increases the risk of subsequent fracture. The history of fragility fractures is an important risk factor for subsequent fractures [2]. Given that osteoporosis is a medical condition which is usually clinically undiagnosed for longer time periods, the ability to determine fracture risk is of paramount importance. Some prognostic tools have been developed, including FRAX [3] and Garvan calculators [4, 5]. These tools are available on the Internet and enable to establish fracture risk (Garvan) or fracture probability limited by life expectancy (FRAX). FRAX measures the probability of hip or major fractures for women and men over a decade, Garvan establishes the risk for hip and any fractures in women and men over 5 and 10 years, and estimates any fracture risk over 5- and 10-year periods only in women. The practical role of such estimations in everyday practice is to identify subjects at increased fracture risk and thus with a need of immediate therapy. Methods, designed to establish fracture risk, were presented and discussed in some reports [6–8]. In general, an early assessment of fracture risk is very important for therapy success and may also be helpful for early therapy initiation. An important point is how precisely the baseline fracture risk assessment will correspond to the actual fracture prevalence values during 5- or 10-year observation periods. In some studies, the problem of precise fracture risk assessment was raised and discussed [9–11]. Several studies compared the FRAX and Garvan tools [12–22] in various populations. In general, the studies, aimed to show the predictive potentials of various fracture risk estimation tools, may be useful for clinical practitioners.

A long-term observation seems to be optimal for the practical verification of the prognostic apparatus of the tools designed to estimate fracture risks. We present results of a longitudinal observation of the epidemiological female sample of the RAC-OST-POL Study, gathered in 2012 [23].

The aim of our study was to establish how precisely the FRAX and Garvan tools predicted hip and major fracture risk (FRAX) and any fracture risk (Garvan) over one decade.

## Material and methods

The study group included a population-based postmenopausal sample from the RAC-OST-POL Study. The detailed clinical characteristics were presented earlier [23]. At baseline, 978 subjects were recruited. The “core” cohort of 625 women, included in the RAC-OST-POL study, was randomly selected from the local population of the Racibórz district. The total number of women at the district was 57 357 and the total population of the qualifiable women (> 55 years), inhabiting the region at the time of enrollment into the study was 17,500. Next, 1750 subjects were randomly selected and invited via regular e-mail exchange to participate in the study. A blind list of women, selected for the study, was provided by the local government and each woman was assigned a number without showing her name. A group of 625 women responded positively to the invitation and declared their intention to take part in the study. Together with the women invited by email, additional 353 female volunteers were also included. We considered that addition as beneficial and increasing the data volume, obtained from a larger population. Prior to that decision, we checked whether the method of recruitment (random or nonrandom) had depended on the prior fracture prevalence. The chi-squared test showed that fracture history did not differ between the groups (33% in the randomized population and 29% in the non-randomly included population, chi-squared value = 1.24, *p* = 0.26). Finally, all the women were combined into a larger group of 978 women as the process of inclusion was not associated with the baseline fracture prevalence. We also compared fracture probabilities obtained by the FRAX tool and the fracture risk from the Garvan calculator nomograms for any fracture risk and hip fracture risk, and the values did not differ in any significant way in regard to the randomized or nonrandomized subcohort.

Each year between 2011 and 2020, all the patients were enquired via phone about any fractures during the previous year. All the data were gathered by one investigator (WP). After the 10-year follow-up, 640 women remained under observation. The total drop-out was then 34.5%, while some subjects were lost to follow-up, due to the following reasons: the loss of contact, 25.6%; death, 7.5%; refusal to cooperate, 1%; and the lack of baseline DXA scans, 0.4%). Table 1 presents the baseline clinical characteristics for the subjects who completed the entire follow-up period. In order to assess whether the drop-out effect could significantly bias the results at the end of follow-up period, the most important clinical features of women, who did not complete the study, were additionally analyzed and compared with the final study group. The women, who were lost to follow-up, were older in comparison to the subjects included in the final analysis (67.4 ± 8.6 vs. 65.0 ± 6.95; *p* < 0.001, respectively). However, they did not differ significantly in terms of the crucial risk factors for fracture, e.g., FN T-score (− 1.34 ± 0.9 vs. − 1.24 ± 0.9; *p* = 0.08), the incidence of fractures before the enrolment into the study (27.2% vs. 30.4%; *p* = 0.30), the incidence of falls during the year preceding enrolment (35.7% vs. 32.4%; *p* = 0.30), and the frequency of steroid use (7.1% vs. 5.0%; *p* = 0.18).

**Table 1:** Clinical characteristics of the whole study group and subgroups with and without fractures during follow-up

Variable	Whole group *n* = 640 Mean ± SD	Women with fractures *n* = 129 Mean ± SD	Women without fractures *n* = 511 Mean ± SD
Age, years	65.04 ± 6.95	66.61 ± 7.22*	64.64 ± 6.83
Height, cm	156.6 ± 5.6	156.8 ± 5.29	156.6 ± 5.7
Weight, kg	74.5 ± 14.0	73.9 ± 12.8	74.6 ± 14.3
BMI, kg/m^2^	30.36 ± 5.43	30.10 ± 5.24	30.42 ± 5.48
Menopause age, years	49.20 ± 4.93	49.27 ± 5.22	49.16 ± 4.86

^*^Significantly higher than in the women without fractures, *p* < 0.01; the other variables did not differ

At baseline, bone densitometry (DXA) was performed at the proximal femur, using a Lunar DPX (GE Healthcare, Madison, WI, USA) device. The coefficients of variation (CV%) for the femoral neck and for total hip were 1.6% and 0.82%, respectively. Based on the obtained DXA results and the gathered information about clinical risk factors, the fracture risk was established, using the FRAX calculator specific for the Polish female population and the Garvan tool. The DXA results and the prevalence of clinical risk factors, recorded at the baseline examination, that influenced the results of fracture risk calculation, is presented in Table 2.

**Table 2:** The DXA results [mean ± SD] and the prevalence [*n* (%)] of clinical risk factors recorded at baseline examination that influence the results of fracture risk calculation in the whole study group and subgroups with and without fracture during follow-up

Risk factor	Whole group *n* = 640	Women with fractures *n* = 129	Women without fractures *n* = 511
Femoral neck T-score	− 1.24 ± 0.92	− 1.46 ± 0.91*	− 1.18 ± 0.92
Previous fracture	195 (30.5%)	58 (45.0%)**	137 (26.8%)
Hip fracture in parents	44 (6.9%)	9 (7.0%)	35 (6.8%)
Smoking	68 (10.6%)	10 (7.8%)	58 (11.3%)
Steroid use	33 (5.2%)	11 (8.5%)	22 (4.3%)
Rheumatoid arthritis	38 (5.9%)	10 (7.8%)	28 (5.5%)
Secondary osteoporosis	30 (4.75)	7 (5.4%)	23 (4.5%)
Alcohol intake	5 (0.8%)	0 (0.0%)	5 (1.0%)
Falls over the last 12 months	207 (32.3%)	56 (43.4%)***	151 (29.5%)

^*^Significantly lower than in the women without fractures, *p* < 0.01

^**^Significantly more frequent than in the women without fractures, *p* < 0.0001

^***^Significantly more frequent than in the women without fractures, *p* < 0.01

During the period of observation, 190 osteoporotic fractures occurred in 129 subjects. Major osteoporotic fractures (at hip, arm, forearm, or spine location) were noted in 97 women. The fractures occurred in the following skeletal sites: forearm, 81; spine, 30; ankle, 25; hip, 15; arm, 13; rib, 9; foot (except of digits), 7; clavicula, 7; and pelvis, 3. Single fractures were recorded in 91 women, whereas 24 women reported 2 fractures, 7 women, 3; 5 women, 4; and 2 women reported 5 fractures.

### Statistics

All the statistical calculations were performed with the use of the Statistica 13.3 software (StatSoft, Tulsa, OK, USA) and of the PQStat v.1.8.2.238 (PQStat Software, Plewiska, Poland; https://pqstat.pl). The mean values and standard deviations were used for the descriptive statistics of continuous variables. The prevalence of qualitative features was presented as the number of subjects with percentage values. The values of sensitivity, specificity, and balanced accuracy (BAcc) were used to compare the predictive powers of the compared fracture risk assessment tools. BAcc was chosen instead of accuracy (Acc), as the analyzed dataset was characterized by a high degree of imbalance [24]. Patients with any fractures accounted for 20.16% of all analyzed subjects, with major fractures 15.16%, and with hip fractures only 2.19%. BAcc, which is the average of sensitivity and specificity, is recommended as more valuable for evaluating imbalanced data than “classic” Acc. To verify the prediction accuracy of the analyzed diagnostic tools, the receiver operating characteristic (ROC) was studied, as well as the area under the curve (AUC) was calculated using of the DeLong method. The alternative cutoffs, determining high or low fracture risks, were established on the basis of ROC curves.

When identifying the cutoff points, different methods were used, including the analysis of the distance from the top left corner and the Youden index *J* calculated as [25, 26]:

$$J={max}_{t}\left({Sensitivity}_{t}+{Specificity}_{t}-1\right),$$

where *t* denotes the threshold value, for which the index is maximal.

Conceptually, the distance from the top left corner is the minimum distance between the upper left corner of a square of side 1, i.e., the place where the sensitivity and specificity are the highest, as well as the point of the ROC curve. This index is calculated by the following formula:

$$\sqrt{{\left(1-Sensitivity\right)}^{2}+{\left(1-Specificity\right)}^{2}.}$$

A *p*-value at a level of 0.05 was regarded as statistically significant.

## Results

**Fig. 1:**
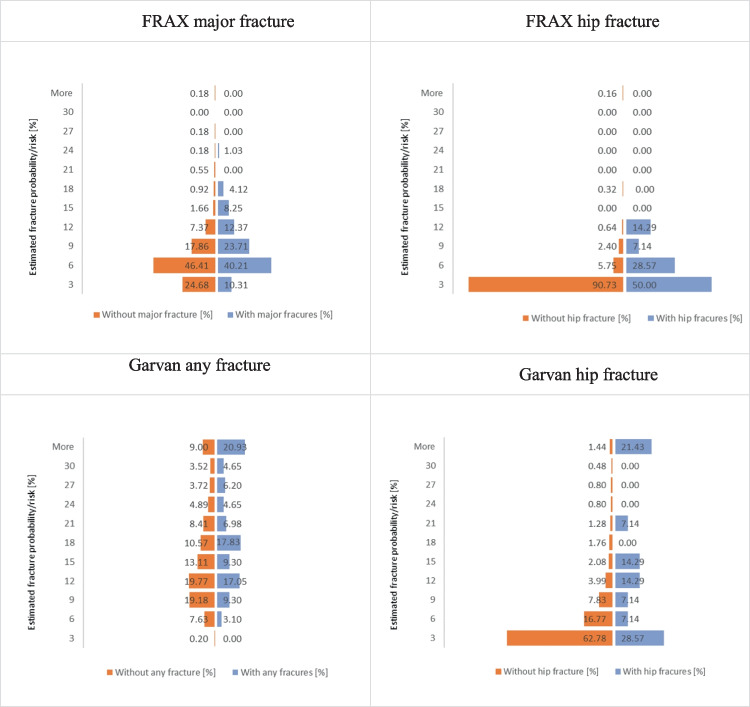
Frequency distributions of FRAX and Garvan risk probabilities for women with and without a fracture

The mean values of 10-year fracture risk/probability, established at baseline for major (FRAX) or any (Garvan) fracture risks and for hip fracture risks (both tools), are presented in Table 3. In addition, the frequency distributions of risk probabilities for the women with and without a fracture, determined for the FRAX and Garvan tools, are presented in Fig. 1.

**Table 3:** The mean values of 10-year fracture risk/probability established at baseline for the compared calculators

Fracture prediction tool	Estimated fracture probability/risk [%] (mean ± SD)
FRAX major fracture	5.63 ± 3.81
FRAX hip fracture	1.42 ± 2.37
Garvan any fracture	17.44 ± 12.62
Garvan hip fracture	4.98 ± 8.87

In order to compare the predictive values of both calculators in relation to the fractures observed during the 10-year follow-up period, all the subjects were first categorized into low or high fracture risk, according to the thresholds of 3% for hip fractures and of 10% for major/any fracture [22, 27]. Table 4 presents information about the number of subjects classified in the high-risk category and the number of observed fractures during the follow-up period.

**Table 4:** The number of subjects classified at high-risk category and the number of observed fractures during follow-up period

Fracture prediction tool	Subjects at high risk (predicted fractures)	Observed fractures (total)	Fractures in high-risk subgroup (predicted and observed)	Fractures in low-risk subgroup (not predicted but observed)
FRAX major fracture	69	97	19 (19.6%)*	78 (80.4%)
FRAX hip fracture	67	14	7 (50.0%)	7 (50.0%)
Garvan any fracture	472	129	111 (86.1%)	18 (13.9%)
Garvan hip fracture	258	14	10 (71.4%)	4 (28.6%)

^*^The percentage values are given in relation to the total number of observed fractures in each category

It is easy to notice that, in the case of the Garvan tool, the vast majority of observed fractures were correctly predicted, while for the FRAX calculator the predictability was much lower, reaching merely 19.59% for major osteoporotic fractures. On the other hand, the Garvan tool showed a much higher fracture prediction power than it was actually observed. The detailed values of the diagnostic indexes for the analyzed predictive tools are provided in Table 5.

**Table 5:** Diagnostics indexes (with 95% CI) for FRAX (major and hip) and Garvan (any and hip) at conventional (≥ 3 and ≥ 10 for hip and major/any fractures, respectively) and newly proposed (≥ 6; ≥ 1.4; ≥ 14.4; and ≥ 8.8, respectively) cutoff points

Fracture prediction tool	Sensitivity	Specificity	BAcc
For conventional (≥ 3 for hip and ≥ 10 for major/any fractures) cutoff points			
FRAX major fracture	0.20 (0.12–0.29)	0.91 (0.88–0.93)	0.55 (0.50–0.61)
FRAX hip fracture	0.50 (0.24–0.76)	0.90 (0.88–0.93)	0.70 (0.56–0.84)
Garvan any fracture	0.86 (0.79–0.91)	0.29 (0.25–0.34)	0.58 (0.52–0.62)
Garvan hip fracture	0.71 (0.42–0.90)	0.60 (0.56–0.64)	0.66 (0.49–0.77)
For newly proposed (≥ 6 for FRAX major, ≥ 1.4 for FRAX hip, ≥ 14.4 for Garvan any, and ≥ 8.8 for Garvan hip) cutoff points			
FRAX major fracture	0.52 (0.41–0.62)	0.70 (0.66–0.74)	0.61 (0.54–0.68)
FRAX hip fracture	0.71 (0.42–0.90)	0.69 (0.65–0.73)	0.70 (0.54–0.81)
Garvan any fracture	0.65 (0.56–0.73)	0.57 (0.53–0.61)	0.61 (0.54–0.67)
Garvan hip fracture	0.64 (0.36–0.86)	0.87 (0.84–0.89)	0.76 (0.60–0.88)

**Fig. 2:**
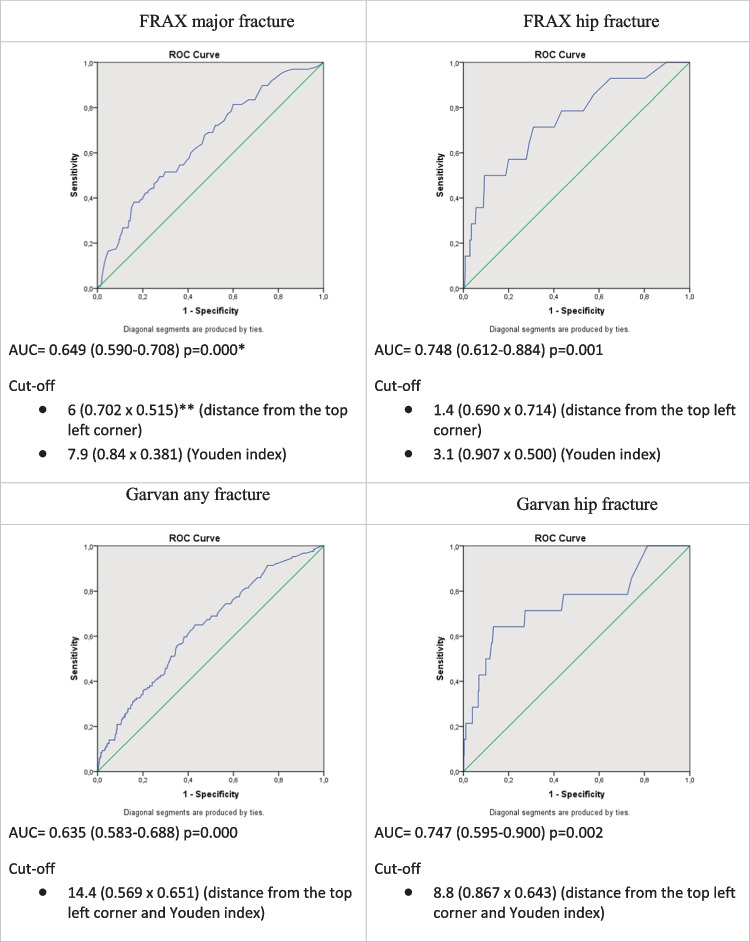
Analysis of receiver operator characteristic (ROC) curves for FRAX and Garvan. *Null hypothesis: true area = 0.5. **The numbers in parentheses indicate specificity and sensitivity values for the proposed cutoff

Considering such a big discrepancy between the diagnostic tools with the same “fixed” cutoff points, resulting in “overestimation” in fracture prediction by the Garvan tool and “underestimation” by FRAX major fracture risks, ROC analyses were additionally performed. They focused on optimal cutoff points, separate for each diagnostic tool, to improve the accuracy of fracture prediction. The achieved ROC curves for each diagnostic tool are presented in Fig. 2.

According to [18, 20], an acceptable model should have both specificity and sensitivity of 50–79%. The following fracture risk threshold values were obtained, corresponding to the optimal diagnostic accuracy: FRAX major fracture risk, 6%; FRAX hip fracture risk, 1.4%; Garvan any fracture risk, 14.4%; and Garvan hip fracture risk, 8.8%. Table 5 shows also the sensitivity, specificity, and balanced accuracy (BAcc) values after the given cutoffs were applied. It may be seen that the BAcc value increased, compared to the values obtained for the previously tested thresholds in all the cases tested.

Hence, the presented analyses confirm the validity of using different cutoff thresholds for both calculators. The relation between the observed fractures and the estimated number of subjects expected to have fractures, predicted on the basis of “new” cutoffs, is presented in Table 6. Compared to the data in Table 4, there is a clear increase in correctly predicted fractures, using the FRAX tool. At the same time, the number of high-risk individuals, predicted by the Garvan tool, decreased while maintaining an acceptable level of correctly predicted fractures (close to that obtained by the FRAX calculator).

**Table 6:** The number of subjects classified at high-risk category with respect to the proposed cutoff values and the number of observed fractures during the follow-up period

Fracture prediction tool	Subjects at high risk (predicted fractures)	Observed fractures (total)	Fractures in high-risk subgroup (predicted and observed)	Fractures in low-risk subgroup (not predicted but observed)
FRAX major fracture (cutoff = 6)	212	97	50 (51.5%)*	47 (48.5%)
FRAX hip fracture (cutoff = 1.4)	204	14	10 (71.4%)	4 (28.6%)
Garvan any fracture (cutoff = 14.4)	304	129	84 (65.1%)	45 (34.9%)
Garvan hip fracture (cutoff = 8.8)	92	14	9 (64.3%)	5 (35.7%)

^*^The percentage values are given in relation to the total number of fractures observed in each category

## Discussion

The predictive potentials of FRAX and Garvan tools are presented in our study via a prospective observation. In general, both tools underestimated the fracture incidence, while the hip fracture rates were more accurately predicted. The Garvan tool better identified therapy demanding subjects than the FRAX calculator. The current study established therapeutic thresholds, helping identify high fracture risk candidates to therapy more accurately than the commonly used thresholds. The thresholds, established in the study and adjusted to the national population, are the most important finding of the current study. We plan to perform an additional longitudinal observation in another, independent population in order to establish the ability of fracture prediction, demonstrated by the FRAX and Garvan tools in comparison to the original local tool, called POL-RISK [28]. We consider that such an external validation may be helpful for practitioners. Such validation should be helpful in accurate identification of subjects who need treatment. In result, a significant number of patients might avoid a fracture.

We consider that the most important issue in management of osteoporotic patients is accurate fracture prediction. Fracture risk depends on several well-defined factors, including the bone status, assessed by bone densitometry, and the so-called clinical risk factors. The crucial aim of osteoporotic therapy is protection against fracture; the primary objective is to prevent the first fracture, while the secondary aim is to avoid the next fracture. These obvious aims are not easy to achieve in daily practice. The number of osteoporotic individuals is rather high and it is not possible to recommend and start treatment in all of them. So, the issue of how to establish precise indications for therapy onset is still open. One should remember that the primary aim is to avoid fracture, but independently of the *purely* medical approach, cost-effectiveness has to be considered as well. In general, the thresholds of 3% for hip and of 20% for any (major) fracture risk are used. In national recommendations, the threshold for the initiation of pharmacological therapy in case of all fracture risk is 10% [27].

In our opinion, the most important point in regard to such clear recommendations is the verification of how the tools, used for fracture risk assessment, predict the real fracture incidence. We should also remember that there are two problems; *first*, how to reveal those individuals who really need treatment, and *second*, how to avoid treatment in patients who in fact are not at high fracture risk. An optimal way to establish the actual values of fracture risk assessment is a prospective observation of a big population plus an analysis of whether fractures really occurred in those who were at high risk at baseline. One should also remember that fractures always occur in a minority of patients. Therefore, the primary aim is to start the therapy in high-risk individuals. The results of the current study enable to establish optimal thresholds for therapy onset, based on specificity and sensitivity. This *pure* medical point of view, which could be a recommendation used in daily practice, should also be approached from the cost-effectiveness perspective. How to find a balance between medical efficacy and economical calculation? Interestingly enough, an application has been proposed by the Garvan tool (www.fractureriskcalculator.com). The thresholds, used to establish therapy initiation points, include the reimbursement of medications. Regarding the hip fracture risk, therapy is recommended when the risk exceeds 9%, and for any fracture risk, it is above 26%. Below 3% and 14%, respectively, the reimbursement of medications is not possible. Between 3 and 9% (hip fracture risk) and 14 and 26% (any fracture risk), the decision should be individualized. The thresholds, proposed in the current study, should be considered as *pure* medical ones. Therefore, an additional analysis is needed in future, taking also into account the therapy costs, in order to establish optimal thresholds for daily clinical practice. The current study shows that the traditional thresholds are used, i.e., 3% for hip fracture risk and 10–20% for any or major fracture risk, it is possible to identify only a small proportion of subjects who need therapy (e.g., those who sustained a fracture during the follow-up period in our study cohort). For the hip, according to the FRAX calculator figures, only 50% women (7 with predicted fracture in comparison to 14 fractures in follow-up) would be treated, and the respective figure for the Garvan tool was 71% (10 versus 14). Regarding the assessment of major fracture risk, according to the FRAX calculator, respective value was 17.6% (17 fractures predicted versus 97 observed) and for any fracture risk, assessed by the Garvan tool, it was 82.2% (106 versus 129). These data indicates that even when the commonly approved thresholds are used, the Garvan tool better identifies the candidates for necessary treatment.

The issue of fracture prediction has been presented in many published reports [14–22]. However, only some of them provide prospective data [14, 15, 18, 19]. In a study of 1422 women, observed for 8.8 years, fracture prediction accuracy was clearly higher for the Garvan tool than for the FRAX calculator [14]. The Garvan tool accurately predicted any fracture risk and overestimated the hip fracture risk. The FRAX calculator underestimated both major and hip fracture risks. The AUC for hip fractures was 0.70 and 0.64 for major fractures (FRAX), and the respective results for the Garvan tool were 0.67 and 0.64. Also, in an Australian observation of 506 women, continued for 5 years, the Garvan tool better predicted fractures than the FRAX tool [15]. The AUCs were 0.693 and 0.689, respectively. In another study, performed with participation of 809 women, observed for 10 years, both the Garvan and FRAX tools underestimated major (any) fracture risk and accurately predicted hip fracture risk [18]. Comparably to our results, the Garvan tool better predicted the later, actual fractures than the FRAX calculator. The ratios for the observed/predicted fractures were 139/184 and 52/115 for the Garvan and FRAX tool, respectively. The AUCs were 0.753 and 0.956 for the FRAX and Garvan tool, respectively. Comparable results were presented in a subsequent study [19], where the FRAX and Garvan tools demonstrated good discriminatory values for hip fracture risk but only a moderate discriminatory ability for major fracture risk (FRAX) and for any fracture risk (Garvan). The respective values of AUC for any (major) fracture risks were 0.708 and 0.721, respectively, and did not significantly differ. The adequate values for hip fracture risk were 0.841 and 0.761, and differed significantly. Regardless of the presented data, the authors showed an interesting analysis of the proportion of the subjects treated and untreated, according to the fracture risk established at baseline: 73.3% (FRAX) and 72.8% (Garvan) of the patients who eventually suffered from a fracture would not have been treated. We believe that the above considerations demonstrate the need to establish other optimal thresholds for therapy onset instead of the to-date’s common thresholds.

The study has certain limitations. We observed only women, some data, obtained via phone interviews, might be incorrect, spine radiograms were not available in all the patients, either at baseline or during follow-up; thus, some vertebral fractures may have been omitted. However, a large epidemiological sample was followed for 10 years and a significant number of fractures were identified, providing valuable results. The new thresholds for therapy onset should enable more patients with high fracture risk to be qualified for treatment.

In conclusion, the described prospective study established new optimal thresholds for the initiation of therapy. The improved identification of patients in need of therapy should decrease new fracture incidence rates.

## Data Availability

The data that support the findings of this study are available from the authors upon reasonable request

## Declarations

A.W. and M.B. participated in the calculations and in the statistical analyses, and identified the appropriate indicators/measures to compare the predictive powers of the compared fracture risk assessment tools, which could be described as the implementation of tasks in the Information and Communication Technology discipline, defined by the Polish Ministry of Education.
